# Long-Term Survival After Transhiatal Versus Transthoracic Esophagectomy: A Population-Based Nationwide Study in Finland

**DOI:** 10.1245/s10434-022-12349-8

**Published:** 2022-08-25

**Authors:** Anna Junttila, Olli Helminen, Mika Helmiö, Heikki Huhta, Raija Kallio, Vesa Koivukangas, Arto Kokkola, Simo Laine, Elina Lietzen, Sanna Meriläinen, Vesa-Matti Pohjanen, Tuomo Rantanen, Ari Ristimäki, Jari V. Räsänen, Juha Saarnio, Eero Sihvo, Vesa Toikkanen, Tuula Tyrväinen, Antti Valtola, Joonas H. Kauppila, the FINEGO Group

**Affiliations:** 1 Division of Digestive Surgery and Urology, Turku University Hospital, Turku, Finland; 2 Surgery Research Unit, Medical Research Center Oulu, Oulu University Hospital and University of Oulu, Oulu, Finland; 3 Department of Oncology and Radiotherapy, Oulu University Hospital, Oulu, Finland; 4 Department of Surgery, University of Helsinki and Helsinki University Hospital, Helsinki, Finland; 5 Cancer and Translational Medicine Research Unit, Medical Research Center Oulu, University of Oulu and Oulu University Hospital, Oulu, Finland; 6 Department of Surgery, University of Eastern Finland and Kuopio University Hospital, Kuopio, Finland; 7 Department of Pathology, HUSLAB, HUS Diagnostic Center, Helsinki University Hospital and University of Helsinki, Helsinki, Finland; 8 Applied Tumor Genomics Research Program, Research Programs Unit, Faculty of Medicine, University of Helsinki, Helsinki, Finland; 9 Department of General Thoracic and Oesophageal Surgery, Heart and Lung Centre, University of Helsinki and Helsinki University Hospital, Helsinki, Finland; 10 Department of Surgery, Central Finland Central Hospital, Jyväskylä, Finland; 11 Department of Cardiothoracic Surgery, Heart Center, Tampere University Hospital and University of Tampere, Tampere, Finland; 12 Department of Gastroenterology and Alimentary Tract Surgery, Tampere University Hospital, Tampere, Finland; 13 Upper Gastrointestinal Surgery, Department of Molecular Medicine and Surgery, Karolinska Insitutet and Karolinska University Hospital, Stockholm, Sweden

## Abstract

**Background:**

No population-based studies comparing long-term survival after transhiatal esophagectomy (THE) and transthoracic esophagectomy (TTE) exist. This study aimed to compare the 5-year survival of esophageal cancer patients undergoing THE or TTE in a population-based nationwide setting.

**Methods:**

This study included all curatively intended THE and TTE for esophageal cancer in Finland during 1987–2016, with follow-up evaluation until 31 December 2019. Cox proportional hazard models provided hazard ratios (HRs) with 95% confidence intervals (CIs) of 5-year and 90-day mortality. The results were adjusted for age, sex, year of operation, comorbidities, histology, neoadjuvant treatment, and pathologic stage.

**Results:**

A total of 1338 patients underwent THE (*n* = 323) or TTE (*n* = 1015). The observed 5-year survival rate was 39.3% after THE and 45.0% after TTE (*p* = 0.072). In adjusted model 1, THE was not associated with greater 5-year mortality (HR 0.99; 95% CI 0.82–1.20) than TTE. In adjusted model 2, including T stage instead of pathologic stage, the 5-year mortality hazard rates after THE (HR 0.87, 95% CI 0.72–1.05) and TTE were comparable. The 90-day mortality rate for THE was higher than for TTE (adjusted HR 0.72; 95% CI 0.45–1.14). In subgroup analyses, no differences between THE and TTE were observed in Siewert II gastroesophageal junction cancers, esophageal cancers, or pN0 tumors, nor in the comparison of THE and TTE with two-field lymphadenectomy. The sensitivity analysis, including patients with missing patient records, who underwent surgery during 1996–2016 mirrored the main analysis.

**Conclusions:**

This Finnish population-based nationwide study suggests no difference in 5-year or 90-day mortality after THE and TTE for esophageal cancer.

**Supplementary Information:**

The online version contains supplementary material available at 10.1245/s10434-022-12349-8.

Esophageal cancer is the sixth leading cause of cancer death worldwide.^1^ In early or locally advanced disease, surgery offers the best chance for cure.^2^ Transhiatal esophagectomy (THE) and transthoracic esophagectomy (TTE) are valid surgical alternatives.^3^ The THE procedure is performed via an abdominal and neck incision,^4^ whereas the TTE procedure includes a thoracotomy/thoracoscopy allowing either intrathoracic or neck anastomosis and more extensive lymphadenectomy.^5^

For frail patients, THE is sometimes preferred to avoid complications,^6^ which have a negative effect on survival^7^ and long-term quality of life.^8^ A British prospective study suggested better quality of life outcomes after THE than after TTE.^9^ However, the long-term oncologic outcomes after THE versus TTE are still debated.

The first two randomized controlled trials comparing THE and TTE indicated no differences in morbidity or survival.^10,11^ Furthermore, a Dutch randomized controlled trial of 220 patients with distal esophageal or gastroesophageal junction adenocarcinoma suggested a borderline better survival after TTE.^12,13^ However, these studies have been criticized for being inadequately powered for survival analysis.

A recent Dutch nationwide propensity score-matched cohort study suggested that TTE is associated with higher morbidity and short-term mortality rates.^14^ Another Dutch study showed equivalent survival after minimally invasive esophagectomy (MIE) compared with open esophagectomy (OE) using either THE or TTE.^15^ A prospective single-center American study suggested improved survival for patients who underwent TTE compared with THE.^16^ However, large, population-based studies comparing long-term survival between THE and TTE are lacking.

This study aimed primarily to examine the 5-year survival of esophageal cancer patients undergoing THE versus TTE. The secondary aim was to compare 90-day mortality between the two approaches.

## Methods

### Study Design

This was a population-based, nationwide, retrospective cohort study from Finland including curatively intended esophagectomy for esophageal adenocarcinoma or squamous cell carcinoma. The study period was from 1 January 1987 to 31 December 2016, with follow-up until 31 December 2019.^17^ As the main outcome, THE was compared with TTE in relation to 5-year all-cause mortality. The study was approved by the Regional Ethical Review Board in Oulu, Finland and by the Finnish national health officials and hospital districts.^18^

### Data Collection

In single-center studies, retrospective comparison of long-term survival after different surgical techniques is prone to bias. The Finnish National Esophago-Gastric Cancer Cohort (FINEGO) includes all patients with esophageal and gastric cancer diagnosed in Finland between 1987 and 2016.^17^ The FINEGO database contains information from the Finnish Cancer Registry, the Finnish National Institute for Health and Welfare registries, and the Care Register for Healthcare and Hospital Discharge Registry. The Finnish Cancer Registry is 92% and the Hospital Discharge Registry 98% complete for esophageal cancer.^19^

Surgically treated patients were identified using the Finnish Hospital League codes until 1995 and the Nordic Medico-Statistical Committee (NOMESCO) surgical codes from 1996 to 2016. Identification using both registries to search for cancer diagnoses and operation codes allows 100% completeness of eligible patient identification.

After identification of cases, available information including age, sex, comorbidity,^20^ surgery, and other variables was collected from the Finnish Cancer Registry, the Finnish National Institute for Health and Welfare registries, the Care Register for Healthcare, and the Hospital Discharge Registry.^17^ Medical reports were obtained from the respective health care units and reviewed by specialized surgeons, providing accurate information on type of resection, tumor location, histology, stage, and neoadjuvant treatment. All-cause mortality data was obtained from the 100% complete death registry held by Statistics Finland until 31 December 2019.^21^

### Exposures

The study exposure was THE (exposure group) with TTE (control group).

### Outcomes

The primary outcome of the study was 5-year all-cause mortality, and the secondary outcome was 90-day all-cause mortality.

### Statistical Analysis

The analyses followed a detailed a priori study protocol. For all analyses, IBM SPSS v26.0 (IBM Corp., Armonk, NY, USA) was used. Follow-up times were calculated from the date of surgery until the time of death or the end of the follow-up period, whichever occurred first. Observed survival was calculated using the life table method, visualized with Kaplan–Meier curves. Cox proportional hazard models provided hazard ratios (HRs) with 95% confidence intervals (CIs) for all mortality outcomes.

To avoid confounding, two models of adjustments were designed for seven known prognostic factors: age (continuous), sex (male/female), year of the surgery (in 3-year groups: 1987–1989, 1990–1992, 1993–1995, 1996–1998, 1999–2001, 2002–2004, 2005–2007, 2008–2010, 2011–2013, 2014–2016), comorbidity (Charlson Comorbidity Index^20^ 0, 1, or ≥ 2, excluding the esophageal cancer under treatment), histologic type of cancer (adenocarcinoma or squamous cell carcinoma, with indirect adjustment for tobacco smoking and alcohol overconsumption), neoadjuvant therapy (yes/no), pathologic stage in model 1 (stages 0–1, 2, 3, 4, according to 8th-edition AJCC/UICC staging of cancers of the esophagus and esophagogastric junction^22^), and T stage (T0, Tis–T1, T2, T3, T4) in model 2 to avoid possible upstaging due to higher lymph node yield after TTE.

Furthermore, analyses were performed for the five following subgroups: (1) patients who underwent surgery during the years 2002–2016, when selection bias due to missing patient records was considerably lower than in the earlier years, (2) patients with tumors located in the proximal to lower esophagus where thoracic lymphadenectomy is considered essential, (3) patients with tumors located in the gastroesophageal junction where abdominal lymphadenectomy is considered more important than thoracic lymphadenectomy, (4) patients who had THE with any lymphadenectomy versus TTE with two-field lymphadenectomy to reduce variability of intrathoracic lymphadenectomy in TTE procedures, and (5) patients with no metastatic lymph nodes (pN0) due to possible upstaging related to more extensive lymphadenectomy in TTE. Adjustments for the subgroups also were performed as described earlier, except for the patients with no lymph node metastases (N0), whose tumor stage was replaced with adjustment for T stage (T0, Tis–T1, T2, T3, T4).

Patients with completely missing medical records or unclear exposure information were excluded from the main analysis. Missing confounder data were handled by performing both complete case analysis and multiple imputation.^23^ The results did not differ between complete case analysis and multiple imputation, so only the imputed results are presented.

In 1996, NOMESCO operation codes allowing differentiation between the exposure groups came into use. To examine whether the patients with missing medical records contributed to the results, a sensitivity analysis was performed including all esophageal cancer patients who underwent surgery during the years 1996–2016, with exclusions described in Fig. S1. Multiple imputation was used to impute TNM8 pathologic stage (0, 1, 2, 3, 4) and pT-stage (T0, Tis–T1, T2, T3, T4) based on data from the cancer registry and medical records and to handle missing data. The adjustments for confounding factors were made as described earlier except that the year of the surgery was divided into four groups (1996–2000, 2001–2005, 2006–2010, 2011–2016).

Subgroup analyses for the THE patients versus the TTE patients with two-field lymphadenectomy alone or the patients with no metastatic lymph nodes (pN0) were not performed because the information was available only for the patients with patient records available.

## Results

### Patients

**Fig. 1:**
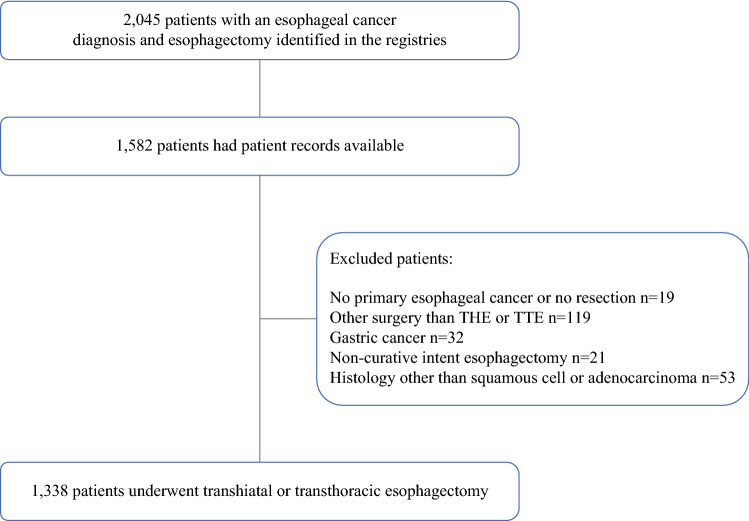
The study population in the main analysis

A total of 2045 patients with an esophageal cancer diagnosis and esophagectomy were identified in the registries. The records for 1582 of these patients were available for analysis, and 1338 patients were included in the study (Fig. 1). Of these 1338 patients, 323 (24.1%) underwent THE, and 1015 (75.9%) underwent TTE. The transthoracic procedures included 715 Ivor-Lewis and 300 McKeown esophagectomies.

The majority of the study patients were men, who had a diagnosis of adenocarcinoma, tumor located in the lower esophagus or gastroesophageal junction, and pathologic stage 3 disease. Neoadjuvant therapy was administered to 35.7% of the study patients. Baseline characteristics stratified by the surgical approach are shown in Table 1. The patients in the THE group more often had surgery during the earlier years of the study, were older, were more frequently female, had neoadjuvant therapy or minimally invasive surgery less often, and had a lower lymph node yield.

**Table 1:** Characteristics of the 1338 esophageal carcinoma patients who underwent transhiatal or transthoracic esophagectomy in Finland from 1987 to 2016

	Whole cohort (*n* = 1338) *n* (%)	Transhiatal esophagectomy (*n* = 323) *n* (%)	Transthoracic esophagectomy (*n* = 1015) *n* (%)
Year of the operation			
1987–1989	78 (5.8)	51 (15.8)	27 (2.7)
1990–1992	74 (5.5)	46 (14.3)	28 (2.8)
1993–1995	91 (6.8)	41 (12.7)	50 (4.9)
1996–1998	114 (8.5)	33 (10.2)	81 (8.0)
1999–2001	114 (8.5)	25 (7.7)	89 (8.8)
2002–2004	120 (9.0)	23 (7.1)	97 (9.5)
2005–2007	132 (9.9)	36 (11.2)	96 (9.5)
2008–2010	172 (12.9)	34 (10.5)	138 (13.6)
2011–2013	198 (14.8)	24 (7.4)	174 (17.1)
2014–2016	245 (18.3)	10 (3.1)	235 (23.1)
Median age: years (IQR)	65 (58–71)	67 (61–74)	64 (57–70)
Sex			
Male	935 (69.9)	208 (64.4)	727 (71.6)
Female	403 (30.1)	115 (35.6)	288 (28.4)
CCI			
0	843 (63.0)	220 (68.1)	623 (61.4)
1	339 (25.3)	68 (21.1)	271 (26.7)
≥2	156 (11.7)	35 (10.8)	121 (11.9)
Tumor histology			
Adenocarcinoma	806 (60.2)	169 (52.3)	637 (62.8)
Squamous cell carcinoma	527 (39.4)	152 (47.1)	375 (36.9)
Missing	5 (0.4)	2 (0.6)	3 (0.3)
Tumor location			
Upper or middle	308 (23.0)	92 (28.5)	216 (21.3)
Lower	678 (50.7)	163 (50.5)	515 (50.7)
Siewert II	343 (25.6)	66 (20.4)	277 (27.3)
Missing	9 (0.7)	2 (0.6)	7 (0.7)
Pathologic stage			
0–1	404 (30.2)	104 (32.2)	300 (29.6)
2	245 (18.3)	63 (19.5)	182 (17.9)
3	506 (37.8)	124 (38.4)	382 (37.6)
4	144 (10.8)	20 (6.2)	124 (12.2)
Missing	39 (2.9)	12 (3.7)	27 (2.7)
N stage			
N0	801 (59.9)	217 (67.2)	584 (57.5)
≥N1	537 (40.1)	106 (32.8)	431 (42.5)
Neoadjuvant treatment			
Yes	478 (35.7)	32 (9.9)	446 (43.9)
No	846 (63.2)	283 (87.6)	563 (55.5)
Missing	14 (1.1)	8 (2.5)	6 (0.6)
Operative approach			
Open	1060 (79.2)	319 (98.8)	741 (73.0)
Hybrid	55 (4.1)	0	55 (5.4)
Minimally invasive esophagectomy	223 (16.7)	4 (1.2)	219 (21.6)
Median lymph node yield (IQR)	11 (3–22)	3 (0–8)	15 (7–26)
Missing	69 (5.2)	20 (6.2)	49 (4.8)
Radicality			
R0	1146 (85.7)	268 (83.0)	878 (86.5)
R1 or R2	178 (13.3)	47 (14.6)	131 (12.9)

*IQR* interquartile range, *CCI* Charlson Comorbidity Index

### Primary Outcomes

**Fig. 2:**
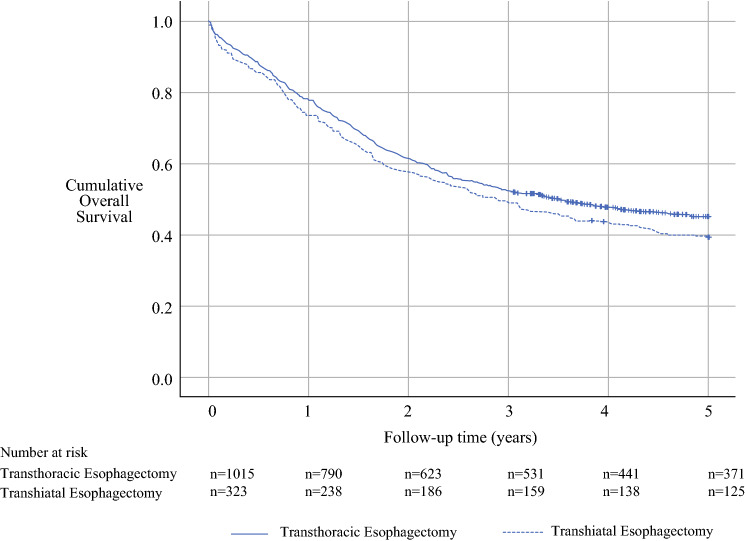
Kaplan–Meier curves showing observed 5-year survival in the main analysis comparing patients the who underwent transhiatal esophagectomy with those who had transthoracic esophagectomy

The observed 5-year survival was 39.3% after THE and 45.0% after TTE (*p* = 0.072, log rank test; Fig. 2). After adjustment for confounding factors, THE was not associated with a higher 5-year all-cause mortality rate than TTE in model 1 (HR 0.98; 95% CI 0.81–1.19; Table 2). In model 2, the point estimate for 5-year all-cause mortality hazard was slightly lower after THE (HR 0.87; 95% CI 0.72–1.05), but the difference was not statistically significant.

**Table 2:** Risk of 5-year mortality after surgery for esophageal cancer compared between transhiatal and transthoracic esophagectomies in the whole cohort and in pre-specified subgroups

	No. of patients	Transhiatal esophagectomy HR (95% CI)	Transthoracic esophagectomy HR (95% CI)
All patients (crude)	1338	1.16 (0.99–1.37)	1.00 (Reference)
All patients (adjusted)^a^	1338	0.98 (0.81–1.19)	1.00 (Reference)
All patients (adjusted)^b^	1338	0.87 (0.72–1.05)	1.00 (Reference)
Patients who had surgery between 2002 and 2016			
All patients (crude)	867	1.10 (0.85–1.42)	1.00 (Reference)
All patients (adjusted)^a^	867	1.27 (0.96–1.68)	1.00 (Reference)
Esophageal excluding Siewert II tumors			
All patients (crude)	992	1.17 (0.97–1.41)	1.00 (Reference)
All patients (adjusted)^a^	992	0.93 (0.75–1.16)	1.00 (Reference)
Gastroesophageal junction (Siewert II) tumors			
All patients (crude)	346	1.08 (0.76–1.56)	1.00 (Reference)
All patients (adjusted)^a^	346	1.05 (0.69–1.60)	1.00 (Reference)
Transhiatal esophagectomy or transthoracic esophagectomy with two-field lymphadenectomy			
All patients (crude)	1170	1.18 (1.00–1.40)	1.00 (Reference)
All patients (adjusted)^a^	1170	0.97 (0.78–1.20)	1.00 (Reference)
pN0 tumors			
All patients (crude)	801	1.48 (1.18–1.86)	1.00 (Reference)
All patients (adjusted)^b^	801	1.02 (0.77–1.34)	1.00 (Reference)

*HR* hazard ratio, *CI* confidence interval

^a^Adjusted for age (continuous), sex, year of the surgery (in 3-year groups), Charlson Comorbidity Index (0, 1, or ≥ 2), histology, neoadjuvant therapy (yes or no), and pathologic stage (0–1, 2, 3, 4)

^b^Adjusted for age (continuous), sex, year of the surgery (in 3-year groups), Charlson Comorbidity Index (0, 1, or ≥ 2), histology, neoadjuvant therapy (yes or no), and T stage (T0, Tis–T1, T2, T3, T4)

For esophageal tumors, excluding Siewert II tumors, the observed 5-year survival rate was 38% after THE and 44.2% after TTE (*p* = 0.085, log rank test; Fig. S2). For gastroesophageal junctional Siewert II tumors, the observed 5-year survival rate was 43.8% after THE and 47.5% after TTE (*p* = 0.624, log rank test; Fig. S3).

The results of the a priori defined subgroup analyses are presented in Table 2. No statistically significant differences between THE and TTE were found in the crude or adjusted models of the subgroups of patients who underwent surgery in 2002–2016 for esophageal tumors, excluding Siewert II tumors, or gastroesophageal junctional Siewert II tumors. When the TTE patients who underwent less than a two-field lymphadenectomy were excluded, THE was associated with a higher risk of 5-year all-cause mortality in the crude model (HR 1.18; 95% CI 1.00–1.40) than TTE, whereas no statistically significant difference was found in the adjusted model (HR 0.97; 95% CI 0.78–1.20). In the crude-model pN0 tumors, THE was associated with a higher risk of 5-year all-cause mortality (HR 1.48; 95% CI 1.18–1.86) than TTE, whereas the adjusted model showed no statistically significant difference (HR 1.02; 95% CI 0.77–1.34).

### Secondary Outcomes

The 90-day observed survival was 89.5% after THE and 92.3% after TTE. In the regression analysis, THE was not associated with a higher risk of 90-day all-cause mortality than TTE (adjusted HR 0.71; 95% CI 0.45–1.13; Table 3).

**Table 3:** Risk of 90-day mortality after surgery for esophageal cancer compared between transhiatal and transthoracic esophagectomies in the whole cohort and in subgroups during 2002–2016

	No. of patients	Transhiatal esophagectomy HR (95% CI)	Transthoracic esophagectomy HR (95% CI)
All patients (crude)	1338	1.39 (0.93–2.08)	1.00 (Reference)
All patients (adjusted)^a^	1338	0.71 (0.45–1.13)	1.00 (Reference)
Patients operated between years 2002 and 2016			
All patients (crude)	867	1.90 (0.93–3.87)	1.00 (Reference)
All patients (adjusted)^a^	867	2.12 (0.95–4.70)	1.00 (Reference)

*HR* hazard ratio, *CI* confidence interval

^a^Adjusted for age (continuous), sex, year of the surgery (in 3-year groups), Charlson Comorbidity Index (0, 1 or ≥ 2), histology, neoadjuvant therapy (yes or no), and pathologic stage (0–1, 2, 3, 4)

The 90-day observed survival rates for THE and TTE were respectively 91.9% and 92.9% for esophageal tumors, excluding Siewert II tumors, and respectively 92.4% and 93.9% for gastroesophageal junctional Siewert II tumors. No statistically significant differences in risk of 90-day all-cause mortality were found between THE and TTE in the subgroup analysis of the patients who underwent surgery between the years 2002 and 2016 (HR 1.90; 95% CI 0.93–3.87 in the crude model and HR 2.12; 95% CI 0.95–4.70 in the adjusted model).

### Sensitivity Analysis

To exclude potential bias resulting from missing patient records, a sensitivity analysis was performed with all 1427 patients identified in the registries who underwent esophagectomy for esophageal cancer from 1996 and onward. After exclusions (Fig. S1), 1268 patients who underwent THE (*n* = 246, 19.4%) or TTE (*n* = 1022, 80.6%) were included in the sensitivity analysis (Table S1).

**Fig. 3:**
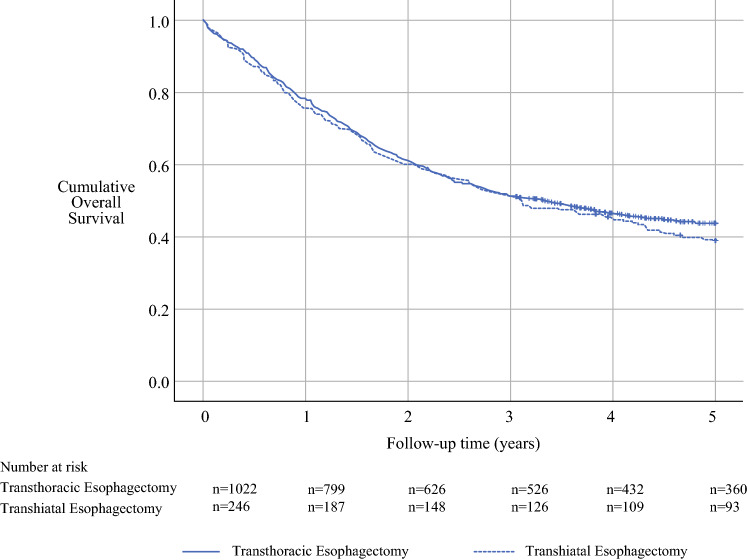
Kaplan–Meier curves showing observed 5-year survival in the sensitivity analysis comparing the patients who underwent transhiatal esophagectomy and those who had transthoracic esophagectomy between the years 1996 and 2016

The observed 5-year survival rate was 38.9% after THE and 43.7% after TTE (*p* = 0.289, log rank test; Fig. 3). For esophageal tumors, excluding Siewert II tumors, the observed 5-year survival rate was 38.9% after THE and 43.0% after TTE (*p* = 0.443, log rank test), and for gastroesophageal junctional Siewert II tumors, it was 38.8% after THE and 45.3% after TTE (*p* = 0.433, log rank test). The THE procedure was not associated with a higher risk of 5-year all-cause mortality than the TTE procedure in model 1 (HR 1.09; 95% CI 0.86–1.38) or model 2 (HR 1.00; 95% CI 0.82–1.24; Table S2).

In the subgroup analysis for esophageal or gastroesophageal junction tumors, no statistically significant differences in risk of 5-year all-cause mortality between THE and TTE were found (Table S2). Neither was THE associated with a greater risk of 90-day all-cause mortality than TTE (adjusted HR 1.05; 95% CI 0.60–1.84; Table S3).

## Discussion

This population-based nationwide cohort study suggests no difference in 5-year or 90-day all-cause mortality between THE and TTE. No statistically significant differences were found in adjusted models or in subgroup analysis between the two approaches. A sensitivity analysis including the patients excluded from the main analysis due to missing patient records for 1996–2016 showed no mortality differences between THE and TTE.

The main strength of this study was its population-based design with complete identification and 100% complete follow-up evaluation of all the study patients with a diagnosis of esophageal cancer in Finland. A certain number of patient records, especially during earlier years, were missing. This, however, was unlikely to cause any selection bias, as reflected by the similarity of results for the subgroup that underwent surgery during 2002–2016, with only a few missing records or data for the complete cohort. Furthermore, a sensitivity analysis that also included patients with missing records who underwent surgery during 1996–2016 yielded results similar to those of the main analysis. The Finnish national registries rely on automatic and independent reporting of diagnosis and procedure codes from the hospitals to the hospital discharge registry and on reports of new cancer cases from clinicians to the Finnish Cancer Registry, making patient identification reliable with high coverage.^19^

The sample size of FINEGO was considered sufficient to enable survival and regression analyses also for smaller subgroups of patients, but study power still was limited in the analysis of 90-day outcomes due to the small number of deaths. However, the observed confidence intervals still contained clinically significant differences in point estimates, and therefore, even larger studies and meta-analyses are needed for confirmation. Due to some missing patient record data during the earlier years of the study, both imputed and nonimputed analyses were performed, showing results similar to those of the main analysis. To reduce the risk of chance findings, the analyses were performed according to a priori study protocol. The results were adjusted for potential confounders, but some unknown confounding or bias may have occurred due to the observational nature of the study.

The THE rate has declined over the years in Finland, which despite adjustment with the year of surgery can cause some confounding. Adjustment with MIE was not possible because very few THE procedures were performed with the minimally invasive route. However, a recent Dutch study showed no differences in survival between minimally invasive and open THE, so this should not be a major confounder.^15^

Although studies have compared short- and long-term outcomes after THE and TTE, the available evidence is contradictory. No previous population-based studies have compared long-term survival between the two approaches. The largest published meta-analysis of both histologic subtypes included 5905 patients (42.6% with THE and 57.4% with TTE) in 52 studies and found no difference in 5-year overall survival.^24^

The most recent meta-analysis including 2331 Siewert II-type adenocarcinoma patients in 11 studies that had survival data available for 1845 patients (45.8% with THE and 54.2% with TTE) suggested a significantly higher 5-year overall survival rate after THE than after TTE.^25^ In contrast, several studies have shown lower survival rates after THE than after TTE.^13,16,26^

The current study showed non-significantly improved 5-year survival after TTE for the total cohort in an unadjusted analysis. However, this study included patients during a long period, and adjustments are crucial to reduction of confounding. An adjusted analysis showed no statistically significant differences in 5-year all-cause mortality between the two techniques. To exclude the effect of upstaging,^27^ a model adjusted for T stage instead of pathologic stage suggested that the 5-year all-cause mortality after THE was at least comparable with that after TTE, supporting the hypothesis that more limited lymphadenectomy might not increase long-term mortality.

The optimal extent of lymphadenectomy during esophagectomy is unclear.^22,28^ Extended lymphadenectomy via the thoracic approach is assumed to be more beneficial with tumors located in the proximal to lower esophagus, whereas abdominal lymphadenectomy with possible lower mediastinal lymphadenectomy is considered sufficient for tumors located in the gastroesophageal junction.^12,29^ It is conceivable that TTE enables direct visualization of the operative field and extended resection of mediastinal lymph nodes,^24–26^ whereas by comparison, the limited view to the mediastinum during THE prevents a full thoracic lymphadenectomy.^24–26^

Several studies have shown a higher lymph node yield after TTE,^15,16,24,26^ which also was seen in the current study. This, however, had no survival effect in any of the analyses, including model 2, which did not adjust for N stage. Because thoracic lymphadenectomy varies within TTE procedures, a subgroup analysis compared all the THE patients with the patients who had TTE and two-field lymphadenectomy alone, but no difference between TTE and THE was observed.

In U.S. hospitals, the 90-day mortality rate for esophagectomies in 15,796 esophageal cancer patients varied from 2.2 to 16.2%.^30^ In the cited population-based study, the observed 90-day mortality was 10.5% after THE and 7.7% after TTE, without a statistically significant difference in the main analysis or any of the subgroup analyses.

In a recent study of 846 patients comparing both open and minimally invasive transthoracic Ivor-Lewis and transhiatal esophagectomies, the 90-day mortality rate was higher with THE (3.0%) than with TTE (1.1%), but the difference was not statistically significant.^16^ Another study of 598 Siewert II-type cancer patients found higher 60-day mortality rates after THE (5.6%) than after TTE (2.3%), but the difference was not statistically significant.^31^

In contrast, studies with higher rates of postoperative morbidity and mortality after TTE than after THE also exist^14,24–26^ especially studies showing similar anastomotic leakage rates^14,32,33^ In a recent propensity score-matched cohort of 766 esophageal cancer patients, TTE showed greater morbidity and 30-day mortality than THE (4.0% vs 1.7%), and the anastomotic leakage rates did not differ.^14^ A single-center study of 828 esophageal cancer patients showed similar anastomotic leakage rates, whereas the proportion of patients with an intrathoracic manifestation of a cervical anastomotic leak was significantly higher after TTE (44% vs 27%).^32^

Regardless of the tumor location, cervical anastomosis has been associated with a higher rate of leakages and recurrent laryngeal nerve injuries.^34,35^ The rate of cervical anastomosis has decreased after implementation of MIE,^36^ but it is used especially for high or mid-esophageal tumors, enabling an adequate free margin.^34^ Anastomotic leakage in the thoracic cavity is considered more severe than leakage in the neck, which also is supported by studies of higher morbidity rates after TTE.^14,32,34^

The existing studies do not show an obvious superiority of THE or TTE in the treatment of operable esophageal cancer. Both THE and TTE have their advantages and disadvantages.^5^ In light of our findings, decreasing the use of THE^36,37^ is not supported considering that survival differences between the two approaches were not observed. A need still exists for other large-scale replication studies to define whether THE and TTE are similarly adequate procedures for the treatment of esophageal cancer. The choice of surgical approach should be made based on the surgeon’s experience and patient-dependent factors.

In conclusion, this population-based nationwide study indicated that THE is not associated with a higher 5-year or 90-day mortality rate than TTE.

## Supplementary Information

Below is the link to the electronic supplementary material.

**Fig. S1** Flowchart of the study population in the sensitivity analysis (Tif 180 Kb)

**Fig. S2** Kaplan–Meier Curves Showing Observed 5-Year Survival Curves Comparing The Patients Who Underwent Transhiatal Esophagectomy With Those Who Had Transthoracic Esophagectomy For Esophageal (Excluding Siewert Ii) Tumors (Tif 132 Kb)

**Fig. S3** Kaplan-Meier curves showing observed 5-year survival curves comparing the patients who underwent transhiatal esophagectomy and those who had transthoracic esophagectomy for gastroesophageal junctional Siewert II tumors. (TIF 132 kb)

Supplementary file 4 (DOCX 15 kb)
