# Supplementary figures and images for: Long-Term Survival After Transhiatal Versus Transthoracic Esophagectomy: A Population-Based Nationwide Study in Finland
Source: Ann Surg Oncol. 2022 Aug 25;29(13):8158–67. doi: 10.1245/s10434-022-12349-8 (PMC9640399; doi:10.1245/s10434-022-12349-8)

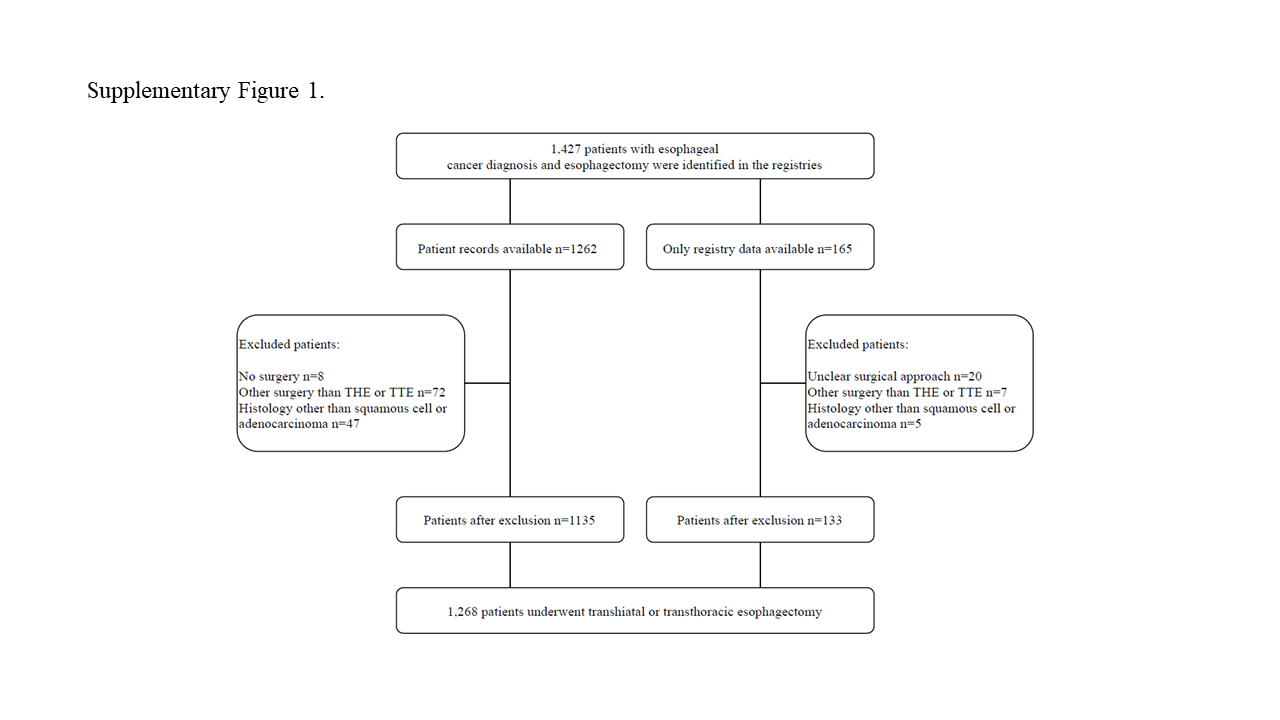

Supplement: Supplementary file 1 — Fig. S1 Flowchart of the study population in the sensitivity analysis (Tif 180 Kb) [file 10434_2022_12349_MOESM1_ESM.tif]

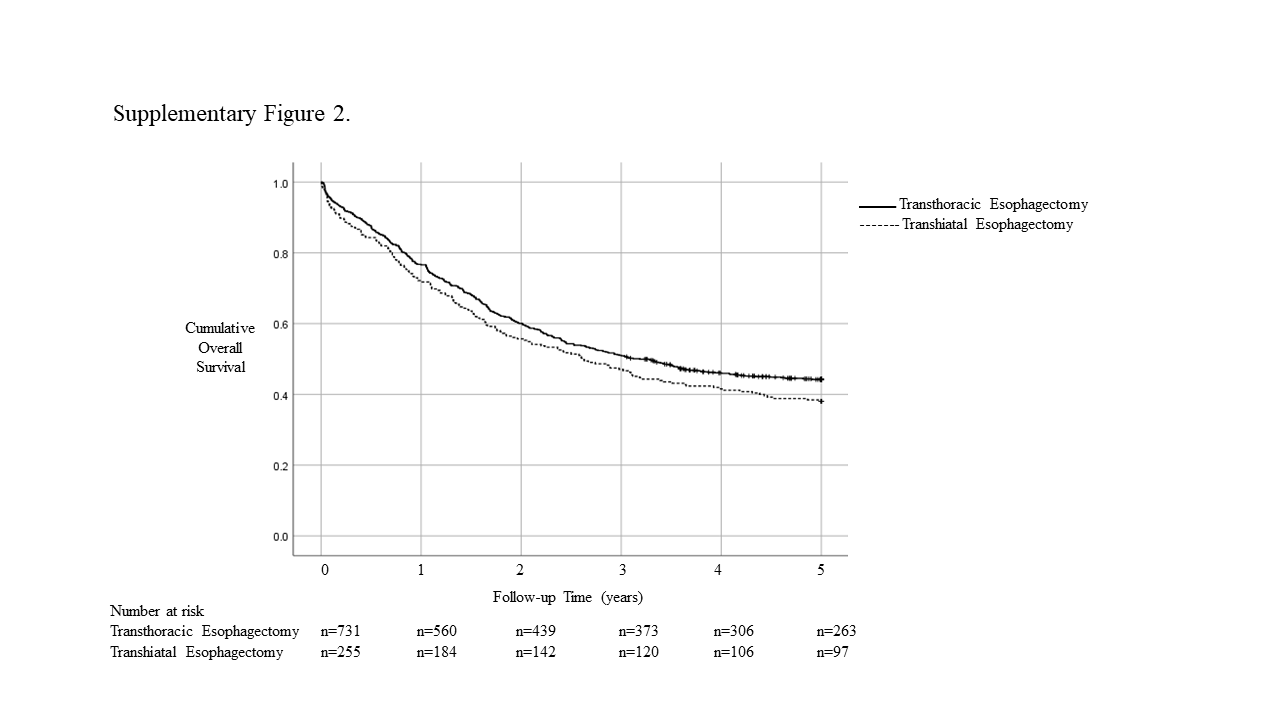

Supplement: Supplementary file 2 — Fig. S2 Kaplan–Meier Curves Showing Observed 5-Year Survival Curves Comparing The Patients Who Underwent Transhiatal Esophagectomy With Those Who Had Transthoracic Esophagectomy For Esophageal (Excluding Siewert Ii) Tumors (Tif 132 Kb) [file 10434_2022_12349_MOESM2_ESM.tif]

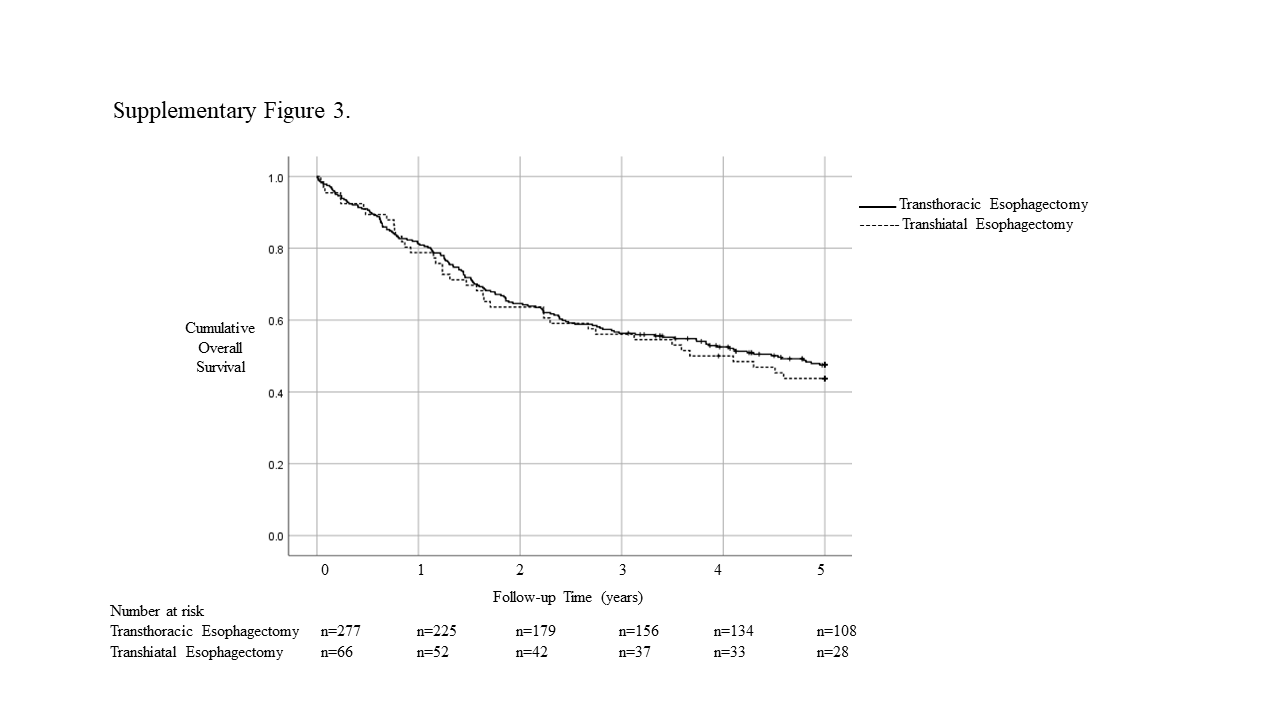

Supplement: Supplementary file 3 — Fig. S3 Kaplan-Meier curves showing observed 5-year survival curves comparing the patients who underwent transhiatal esophagectomy and those who had transthoracic esophagectomy for gastroesophageal junctional Siewert II tumors. (TIF 132 kb) [file 10434_2022_12349_MOESM3_ESM.tif]
