# Supplementary material for: Long-Term Survival After Transhiatal Versus Transthoracic Esophagectomy: A Population-Based Nationwide Study in Finland
Source: Ann Surg Oncol. 2022 Aug 25;29(13):8158–67. doi: 10.1245/s10434-022-12349-8 (PMC9640399; doi:10.1245/s10434-022-12349-8)
Supplement: Supplementary file 4 — (DOCX 15 kb) [file 10434_2022_12349_MOESM4_ESM.docx]

**Supplementary Table 1.** Characteristics of the 1,268 esophageal carcinoma patients operated with transhiatal or transthoracic esophagectomy in Finland 1996 to 2016.

|  | **Whole cohort**  **n=1268** | **Transhiatal esophagectomy**  **n=246** | **Transthoracic esophagectomy**  **n=1022** |
| --- | --- | --- | --- |
| **Year of the operation, n (%)** |  |  |  |
| 1996-2000 | 255 (20.1) | 77 (31.3) | 178 (17.4) |
| 2001-2005 | 283 (22.3) | 70 (28.5) | 213 (20.8) |
| 2006-2010 | 271 (21.4) | 64 (26.0) | 207 (20.3) |
| 2011-2016 | 459 (36.2) | 35 (14.2) | 424 (41.5) |
| **Age, median, IQR** | 65 (58-71) | 67 (59-74) | 64 (58-70) |
| **Sex, n (%)** |  |  |  |
| Male | 925 (72.9) | 170 (69.1) | 755 (73.9) |
| Female | 343 (27.1) | 76 (30.9) | 267 (26.1) |
| **Charlson Comorbidity Index, n (%)** |  |  |  |
| 0 | 758 (59.8) | 140 (56.9) | 618 (60.5) |
| 1 | 347 (27.4) | 73 (29.7) | 274 (26.8) |
| ≥2 | 163 (12.9) | 33 (14.3) | 130 (12.7) |
| **Tumor histology, n (%)** |  |  |  |
| Adenocarcinoma | 837 (66.0) | 168 (68.3) | 669 (65.5) |
| Squamous cell carcinoma | 424 (33.4) | 76 (30.9) | 348 (34.1) |
| Missing | 7 (0.6) | 2 (0.8) | 5 (0.4) |
| **Tumor location, n (%)** |  |  |  |
| Upper or middle | 219 (17.3) | 36 (14.6) | 183 (17.9) |
| Lower | 654 (51.6) | 131 (53.3) | 523 (51.2) |
| Siewert II | 395 (31.2) | 79 (32.1) | 316 (30.9) |
| **Pathological stage, n (%)** |  |  |  |
| 0-I | 361 (28.5) | 72 (29.3) | 289 (28.3) |
| II | 190 (15.0) | 29 (11.8) | 161 (15.8) |
| III | 429 (33.8) | 73 (29.7) | 356 (34.8) |
| IV | 128 (10.1) | 15 (6.1) | 113 (11.1) |
| Missing | 160 (12.6) | 57 (23.2) | 103 (10.0) |
| **Neoadjuvant treatment, n (%)** |  |  |  |
| Yes | 512 (40.4) | 37 (15.0) | 475 (46.5) |
| No | 718 (56.6) | 196 (79.7) | 522 (51.1) |
| Missing | 38 (3.0) | 13 (5.3) | 25 (2.4) |
| **Radicality** |  |  |  |
| R0 | 999 (78.8) | 172 (69.9) | 827 (80.9) |
| R1 or R2 | 127 (10.0) | 19 (7.7) | 108 (10.6) |
| Missing | 142 (11.2) | 55 (22.4) | 87 (8.5) |

**Supplementary Table 2.** Risk of 5-year mortality after surgery for esophageal cancer patients operated between years 1996-2016 comparing transhiatal and transthoracic esophagectomy, expressed as hazard ratios (HR) with 95% confidence intervals (CI).

|  | **Number of patients** | **Transhiatal esophagectomy**  **HR (95% CI)** | **Transthoracic esophagectomy**  **HR (95% CI)** |
| --- | --- | --- | --- |
| **All patients (crude)** | 1268 | 1.10 (0.92-1.32) | 1.00 (Reference) |
| **All patients (adjusted)*** | 1268 | 1.09 (0.86-1.38) | 1.00 (Reference) |
| **All patients (adjusted)**** | 1268 | 1.00 (0.82-1.24) | 1.00 (Reference) |
|  |  |  |  |
| **Esophageal (excluding Siewert II) tumors** |  |  |  |
| **All patients (crude)** | 873 | 1.09 (0.88-1.35) | 1.00 (Reference) |
| **All patients (adjusted)*** | 873 | 1.03 (0.80-1.34) | 1.00 (Reference) |
|  |  |  |  |
| **Gastroesophageal junction (Siewert II) tumors** |  |  |  |
| **All patients (crude)** | 395 | 1.14 (0.83-1.57) | 1.00 (Reference) |
| **All patients (adjusted)*** | 395 | 1.17 (0.77-1.77) | 1.00 (Reference) |

* Adjusted for age (continuous), sex, year of the surgery (four 5-year groups), Charlson Comorbidity Index (0, 1 or ≥2), histology, neoadjuvant therapy (yes or no) and pathological stage (0-I, II, III, IV).

** Adjusted for age (continuous), sex, year of the surgery (four 5-year groups), Charlson Comorbidity Index (0, 1 or ≥2), histology, neoadjuvant therapy (yes or no) and T-stage (T0, Tis-T1, T2, T3, T4).

**Supplementary Table 3.** Risk of 90-day mortality after surgery for esophageal cancer patients operated between years 1996-2016 comparing transhiatal and transthoracic esophagectomy expressed as hazard ratios (HR) with 95% confidence intervals (CI).

|  | **Number of patients** | **Transhiatal esophagectomy**  **HR (95% CI)** | **Transthoracic esophagectomy**  **HR (95% CI)** |
| --- | --- | --- | --- |
| **All patients (crude)** | 1268 | 1.19 (0.70-2.01) | 1.00 (Reference) |
| **All patients (adjusted)*** | 1268 | 1.05 (0.60-1.84) | 1.00 (Reference) |

* Adjusted for age (continuous), sex, year of the surgery (four 5-year groups), Charlson Comorbidity Index (0, 1 or ≥2), histology, neoadjuvant therapy (yes or no) and pathological stage (0-I, II, III, IV).
